# Atypical Choroid Plexus Tumor of the Cauda Equina With Metastases to the Spinal Cord and Brain

**DOI:** 10.7759/cureus.64947

**Published:** 2024-07-19

**Authors:** Tanvir Rizvi, Mohamed Z Hussein, Martha Quezado, Camilo E Fadul, Beatriz Lopes

**Affiliations:** 1 Radiology and Medical Imaging, University of Virginia School of Medicine, Charlottesville, USA; 2 Diagnostic Radiology and Nuclear Medicine, Rush University Medical Center, Chicago, USA; 3 Pathology, National Cancer Institute, Bethesda, USA; 4 Neuro-Oncology, University of Virginia School of Medicine, Charlottesville, USA; 5 Neuropathology, University of Virginia School of Medicine, Charlottesville, USA

**Keywords:** methylation, cranial nerves, spinal cord, cauda equina, choroid plexus tumor

## Abstract

We report a case of a 57-year-old man with a tumor arising from the cauda equina with spinal cord and intracranial metastases in the basal cisterns and along the cranial nerves. He presented with severe lower back pain and mild gait imbalance. His imaging revealed a large mass in the lumbosacral region with involvement of the cauda equina, intradural extramedullary enhancing metastases in the thoracic spinal canal, and intracranial metastases in the suprasellar cistern and along both trigeminal and facial/vestibulocochlear nerve complexes. Pathological examination of the resected thoracic spinal cord mass showed an atypical papillary proliferation with moderate nuclear pleomorphism and rare mitotic figures. While the morphologic and immunophenotypic features were consistent with the diagnosis of a choroid plexus tumor, the atypical location for this entity required the exclusion of other epithelioid tumors with papillary architecture. Additional immunohistochemical markers were used to exclude a metastatic adenocarcinoma, a papillary variant of a meningioma, and a papillary variant of an ependymoma. Ultimately, methylation-based tumor profiling determined that the methylation class was a match for “plexus tumor” resulting in the integrated diagnosis of the tumor with features of choroid plexus papilloma. This is a unique presentation for both the location and the metastatic spread. The methylation profile was instrumental in establishing this diagnosis.

## Introduction

Choroid plexus tumors (CPTs) are rare, representing less than 1% of all brain tumors [1] and 2%-5% of all pediatric tumors [2]. They usually arise in locations where the normal choroid plexus resides- within the ventricular system and are classified according to their histology as choroid plexus papillomas (CPPs), atypical CCP (aCPPs), and choroid plexus carcinomas (CPC). Although these tumors are histologically similar to normal choroid plexus, they can be differentiated from normal tissue by hypercellularity and cellular pleomorphism [3]. Recently they have been classified into three classes according to their methylation profile that correlated with age of appearance, location, and prognosis [4]. 

We report a case of an adult with an atypical CPP arising from the cauda equina with spinal cord and intracranial dissemination. There is only one other case reported of a CPP located in the cauda equina [5], and it did not have craniospinal axis dissemination. This paper mentioned two similar cases in the sacral canal and sacral nerve roots, which were distal to the cauda equina. Our case underscores the evolving role that methylation profiling plays in establishing a more accurate diagnosis and better prognostic estimation of central nervous system tumors.

## Case presentation

A 57-year-old man presented to another facility with a history of subacute low back pain radiating into his right hip, posterolateral calf, sole and lateral aspect of the foot, and mild gait imbalance. He received oral steroids and physical therapy without improvement.

**Figure 1:**
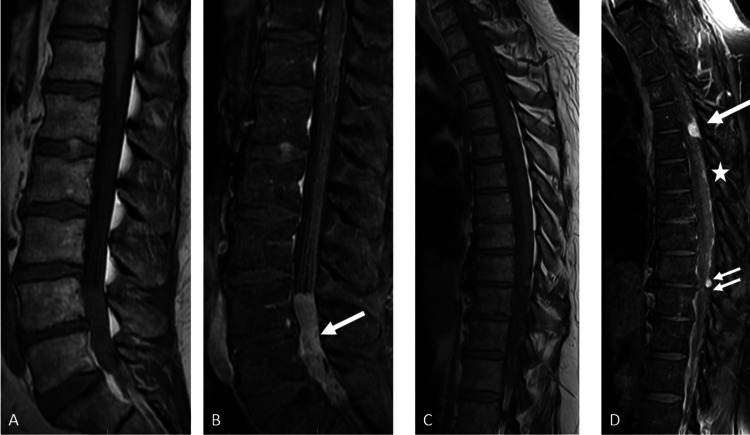
Sagittal T1 pre (A) and post-contrast fat-saturated (B) images of the lumbar spine. Thoracic spine sagittal T1 pre (C) and post-contrast sagittal T1 fat-saturated (D) images. Lumbar spine shows intradural, extramedullary avidly enhancing mass spanning from L4 to S2 (A, B; arrow). Multiple foci of enhancing intradural, extramedullary lesions, largest at T4-T5 (arrow) and T9-T10 disc space levels (C, D; two smaller arrows). Multiple focal enhancing lesions along the surface of spinal cord predominantly dorsally are seen spanning from T6 to T9 levels (star).

A lumbar magnetic response imaging (MRI) study revealed a 7 x 1.5 x 1.3 centimeter (Craniocaudal, anteroposterior and transverse dimension respectively) intradural avidly enhancing mass spanning from L4 to S2 involving nerve roots of the cauda equina (Figures 1A, 1B). The mass was T1 hypointense and T2 intermediate to high signal intensity. He underwent a lumbar laminectomy from the inferior L3 to L5 levels at an outside institution. The mass was encasing the cauda equina nerve roots and only partially removed. The outside pathology diagnosis was of fibrous meningioma with dense tissue and bland spindle cells.

He was referred to our institution with progressive lower back pain radiating into his right leg associated with left foot pain and numbness. He had no weakness or sphincter-related symptoms. His physical examination revealed reduced sensation to light touch in the right posterolateral calf, lateral aspect, and sole of the foot, allodynia of both calves and mild ataxia. MRI studies of the brain and total spine at our institution revealed, in addition to the lumbosacral mass, multiple intradural extramedullary enhancing lesions in the thoracic spine, the largest at right posterolateral aspect at T4-T5 and T9-T10 level (Figures 1C, 1D).

**Figure 2:**
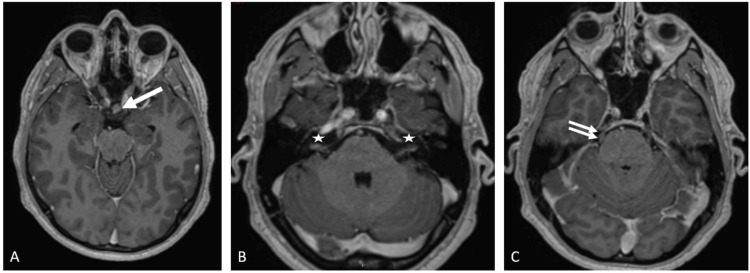
MRI brain axial T1 MPRAGE post-contrast images showing enhancing nodules in suprasellar cistern (A, arrow), bilateral VII-VIII nerve complex (B, stars), and trigeminal nerves (C, two small arrows along right trigeminal nerve).

The T4-T5 lesion was compressing and displacing the cord anteriorly and to the left without cord signal changes. The cervical spine was normal. The brain MRI showed ill-defined enhancing lesions in the suprasellar cistern, along both the trigeminal and the facial/vestibulocochlear nerve complex course (Figures 2A-2C). CT of the chest, abdomen and pelvis did not show any primary malignancy.

**Figure 3:**
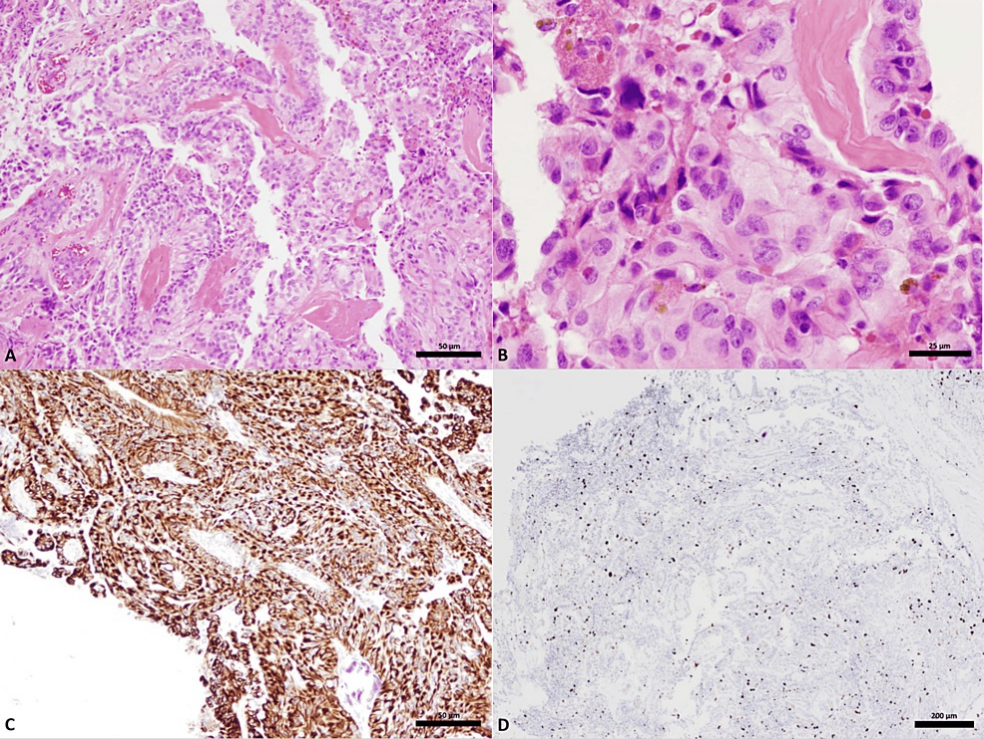
Microscopic features. A low-power view (50 µm) of a hematoxylin and eosin (H&E)-stained slide (A); a high-power view (25 µm) of a H&E-stained slide (B); an immunohistochemical stain for pancytokeratin at low power (50 µm) (C); and an immunohistochemical stain for Ki-67 (200 µm) (D). (A) Atypical papillary proliferation composed of a single to multi-layered epithelioid cells organized around thick fibrovascular cores. (B) Cells are polygonal to columnar with moderate nuclear pleomorphism, occasional prominent nucleoli, rare mitotic figures and focal hemosiderin deposition. (C) Strong diffuse positivity supporting epithelial differentiation. (D) Elevated proliferation index.

The patient had resection of the large thoracic extramedullary spinal lesion at T4-T5 via a posterior laminectomy approach. The tumor composition was grey in color and soft and noted to be adhering to the spinal cord surface. The en bloc tumor removed was sent for pathology review. Microscopic examination of the tumor demonstrated an atypical papillary proliferation composed of single to multi-layered epithelioid cells with underlying thick fibrovascular cores (Grade 2 WHO classification). The cells were polygonal to columnar with moderate nuclear pleomorphism and occasional nucleoli. Rare mitotic figures were seen. The tumor did not show necrosis, microvascular proliferation, and solid areas. There was focal calcification and hemosiderin deposition. The tumor cells were strongly and diffusely positive for pancytokeratin, S-100 protein, and vimentin. There was a membranous staining pattern for EMA. There was focal positivity for GFAP, but the tumor was negative for Oligodendrocyte transcription factor (Olig-2). There was positivity for CD56 and CD99. The tumor cells were focally positive for CK7, negative for CK20, CDX2, GATA-3, thyroid transcription factor (TTF-1), progesterone receptor (PR), SRY-related HMG-box (SOX-10), and mutant BRAF (V600E). A Ki-67 showed an elevated proliferative index (Figures 3A-3D).

**Figure 4:**
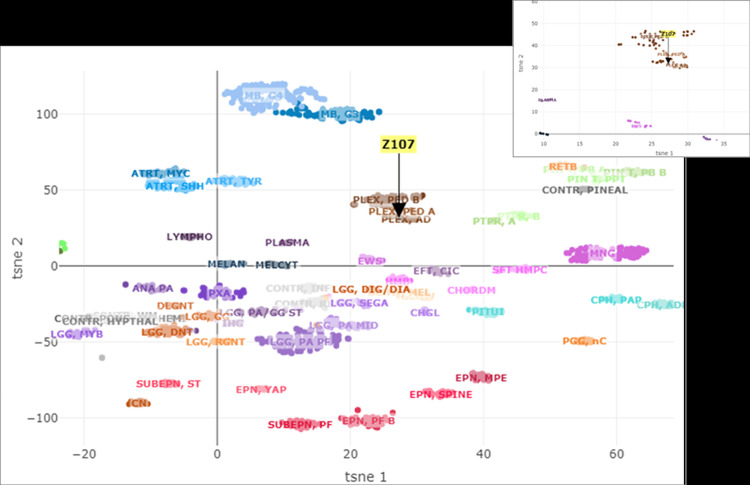
Representation of t-distributed stochastic neighbor embedding (tSNE) of the brain tumor classifier cohort Representation of t-distributed stochastic neighbor embedding (tSNE) of the brain classifier cohort together with the present tumor (Z107) matching with the methylation class plexus tumor, subclass adult.

Based on the morphologic and immunohistochemical features the differential diagnosis included a papillary ependymoma with elevated Ki-67 proliferative index, a metastatic moderately differentiated adenocarcinoma (unlikely given the patient's history and the immunohistochemical profile of the tumor), a papillary variant of a meningioma (unlikely given negative staining for PR) and a choroid plexus neoplasm. The pathologic diagnosis was ultimately a descriptive one and consistent with a papillary epithelioid neoplasm with an elevated Ki-67 proliferative index. Molecular characterization by TruSight 170 Tumor Next Generation Sequencing (Illumina, San Diego, CA, USA) showed no clinically significant variants. The tumor was subsequently analyzed for methylation-based tumor profiling that showed a match with the methylation class plexus tumor, subclass adult (Figure 4) [6]. Therefore, the histological and methylation features supported the diagnosis of an atypical choroid plexus neoplasm.

He underwent proton radiation therapy to the craniospinal axis over six weeks receiving 33 fractions at 180 cGy per fraction. On follow-up eight months after the thoracic surgery, he remains clinically stable and neuro-axis imaging revealed no recurrence in the thoracic spine at T4-T5 level and unchanged residual mass in the caudal aspect of thecal sac at L4-S2 level and spinal cord and intracranial metastases.

## Discussion

Our case represents a choroid plexus tumor of the cauda equina with metastases to the spinal cord and brain, a not previously described feature. Based on the imaging we did not include choroid plexus tumor in the differential diagnosis because of the location of the primary tumor in the cauda equina. Due to the atypical location, the pathology diagnosis was challenging despite the typical histological characteristics of the choroid plexus tumor, although we considered this entity in the differential diagnosis. Because of the unique and atypical imaging and histopathology characteristics of this case, we could only establish the diagnosis by the tumor methylation profile.

CPPs usually occur in proportion to the amount of choroid plexus present in different locations. Approximately 50% arise from the atrium of the lateral ventricle, the left side more common than the right side; 40% arise from the posterior medullary velum and foramina of Luschka of the fourth ventricle; 5% arise from the third ventricle and 5% are in multiple locations [5]. A primary CPP of the spinal canal is exceedingly rare [5-8] with most reported cases of CPT involving the spinal canal being the result of metastases from intraventricular tumors [9-11]. The three case reports of sacral CPPs described well-defined masses amenable to gross total resection without evidence of recurrence on a follow-up of nine months to three years [5-8]. One of these cases had questionable cord enhancement at the T11-T12 level [5]. In all of these cases, the diagnosis of CPP was favored with the microscopic characteristics, but it was a diagnosis of exclusion. The spinal cord and brain dissemination in our case constitutes a not previously described feature.

Methylation profiling of 29 CPPs (WHO grade I), 32 aCPPs (WHO grade II), and 31 CPCs (WHO grade III) was described by Thomas et al. [4]. Unsupervised hierarchical clustering identified three subgroups: cluster 1 (plexus tumor, subclass pediatric A), cluster 2 (plexus tumor, subclass adult), and cluster 3 (plexus tumor, subclass pediatric B). In methylation cluster 3, progression-free survival (PFS) accounted for a mean of 72 months (CI, 55-89 months), whereas only one of 42 tumors of methylation clusters 1 and 2 progressed (P<0.001). Within this classification, our case fits the profiling of a methylation cluster 2 [4]. The epigenetic subgroups pediatric A and adult have CPP and aCPP in histopathology, while subgroup pediatric B has CPP, aCPP, and CPC in the histopathology. The tumor location is supratentorial for subgroup pediatric A and B and infratentorial for subgroup adult. Most importantly, the clinical outcome is high risk for subclass pediatric B and low risk for pediatric A and adult subgroups.

Combining the DNA methylation profile with the mutational status of TP53 may better predict the clinical outcomes [12]. CPCs with homozygous TP53 mutations clustered as a group separate from those carrying a heterozygous TP53 mutation or CPCs with wild-type TP53 and showed the worst survival outcome. Another study suggested that epigenetic profiling of CPTs may provide additional prognostic information in comparison to histopathological grading and can be used for the identification of patients at risk of recurrence [13]. These publications suggest that epigenetic profiling of CPT provides additional prognostic information in comparison to histopathological grading and may be a diagnostic tool in cases where histopathology and location cannot confidently assure the diagnosis. In our case, the methylation profile clustered with the adult CPP and aCPP of mainly infratentorial location (cluster 2) and had wild-type p53 immunoexpression. The patient has had no evidence of progression eight months after surgery. 

Despite the unusual site of occurrence of the present case, the integrated pathological diagnosis is consistent with a papillary neoplasm suggestive of a choroid plexus tumor. The putative cells of origin are ectopic choroid plexus cells, which likely explain the presence of CPT with no connection to the ventricular system. To the best of our knowledge, this is the second case of an adult male with a CPT arising from the cauda equina, and the only case with spinal cord and intracranial dissemination.

## Conclusions

Although rare, this diagnosis of atypical choroid plexus tumor should be considered in the differential diagnosis of masses involving the cauda equina nerve roots. Methylation profiling has diagnostic and prognostic implications in such unusual cases in addition to the role of histopathology and immunohistochemistry in the final diagnosis.
